# Frequency of pharmacogenomic variants affecting efficacy and safety of anti-cancer drugs in a south Asian population from Sri Lanka

**DOI:** 10.1186/s12920-024-01919-2

**Published:** 2024-05-24

**Authors:** Priyanga Ranasinghe, Nirmala Sirisena, Thuwaragesh Vishnukanthan, J. N. Ariadurai, Sathsarani Thilakarathne, C. D. Nelanka Priyadarshani, D. P. Bhagya Hendalage, Vajira H. W. Dissanayake

**Affiliations:** 1 Department of Pharmacology, Faculty of Medicine, University of Colombo, Colombo 08, Sri Lanka; 2 Department of Anatomy, Genetics and Biomedical Informatics, Faculty of Medicine, University of Colombo, Colombo 8, Sri Lanka; 3 University/British Heart Foundation Centre for Cardiovascular Science, The University of Edinburgh, Edinburgh, United Kingdom

**Keywords:** Anti-cancer, Oncology, Pharmacogenomics, *CYP2D6*, *DPYD*, *NUDT15*, *EPAS1*, *XRCC1*, Sri Lanka, South Asia

## Abstract

**Background:**

Therapy with anti-cancer drugs remain the cornerstone of treating cancer. The effectiveness and safety of anti-cancer drugs vary significantly among individuals due to genetic factors influencing the drug response and metabolism. Data on the pharmacogenomic variations in Sri Lankans related to anti-cancer therapy is sparse. As current treatment guidelines in Sri Lanka often do not consider local pharmacogenomic variants, this study aimed to explore the diversity of pharmacogenomic variants in the Sri Lankan population to pave the way for personalized treatment approaches and improve patient outcomes.

**Methods:**

Pharmacogenomic data regarding variant-drug pairs of genes *CYP2D6*, *DPYD, NUDT15, EPAS1*, and *XRCC1* with clinical annotations labelled as evidence levels 1A-2B were obtained from the Pharmacogenomics Knowledgebase database. Their frequencies in Sri Lankans were obtained from an anonymized database that was derived from 541 Sri Lankans who underwent exome sequencing at the Human Genetics Unit, Faculty of Medicine, University of Colombo. Variations in *DPYD*, *NUDT15*, and *EPAS1* genes are related to increased toxicity to fluoropyrimidines, mercaptopurines, and sorafenib respectively. Variations in *CYP2D6* and *XRCC1* genes are related to changes in efficacy of tamoxifen and platinum compounds, respectively. Minor allele frequencies of these variants were calculated and compared with other populations.

**Results:**

MAFs of rs1065852 c.100 C > T (*CYP2D6*), rs3918290 c.1905 + 1G > A (*DPYD*), rs56038477 c.1236G > A (*DPYD*), rs7557402 c.1035–7 C > G (*EPAS1*), rs116855232 c.415 C > T (NUDT15*3), and rs25487 c.1196 A > G (*XRCC1*) were: 12.9% [95%CI:10.9–14.9], 1.5% [95%CI:0.8–2.2], 1.2% [95%CI:0.5–1.8], 37.7% [95%CI:34.8–40.6], 8.3% [95%CI:6.7–10.0], and 64.0% [95%CI:61.1–66.8], respectively. Frequencies of rs1065852 c.100 C > T (*CYP2D6*), rs7557402 c.1035–7 C > G (*EPAS1*), and rs25487 (*XRCC1*) were significantly lower in Sri Lankans, while frequencies of rs116855232 c.415 C > T (NUDT15*3) and rs56038477 c.1236G > A (*DPYD*) were significantly higher in Sri Lankans when compared to some Western and Asian populations.

**Conclusion:**

Sri Lankans are likely to show lower toxicity risk with sorafenib (rs7557402 c.84,131 C > G) and, higher toxicity risk with fluoropyrimidines (rs56038477 c.1236G > A) and mercaptopurine (rs116855232 c.415 C > T), and reduced effectiveness with tamoxifen (rs1065852 c.100 C > T) and platinum compounds (rs25487). These findings highlight the potential contribution of these genetic variations to the individual variability in anti-cancer dosage requirements among Sri Lankans.

## Background

Cancer is a leading cause for morbidity and mortality in the world. According to the World Cancer Research Fund, around 18 million new cases were diagnosed globally in the year 2020 alone [1]. At present a wide variety of treatment modalities are available, including radiation therapy, immunotherapy, endocrine therapy, and gene therapy, while treatment with anti-cancer drugs remains the most widely used modality. Anti-cancer drugs are designed to target and inhibit the growth of cancer cells. A wide range of anti-cancer drugs are currently available, ranging from traditional cytotoxic agents to targeted therapies and immunotherapies, each with unique mechanisms of actions [2].

A large inter-individual variation can be observed among the anti-cancer drugs in terms of efficacy and safety, due to variations in the genes that are involved in the pharmacodynamics and pharmacokinetics of these drugs [3]. For instance, genes encoding CYP2D6 enzyme responsible for phase 1 drug metabolism show genetic polymorphism with resultant variations in enzymatic activity. CYP2D6 is vital in converting anti-cancer drug tamoxifen to its active form. Hence individuals with poor CYP2D6 activity will experience therapeutic failure while those with increased activity will experience adverse effects and toxicities [4, 5]. Numerous other examples exist where efficacy and safety of anti-cancer drugs have been linked to the presence of pharmacogenomic variants present in the patient. Therefore, understanding these genetic variants will provide the opportunity to optimize patient care by using personalized therapy selection and dosing for individual patients.

The study of the relationships between an individual’s or population’s genetic variations and the variable response to the medications is called pharmacogenomics [6]. While substantial research has been conducted on diverse populations worldwide, there is a critical need to explore the pharmacogenomic variations responsible for efficacy and safety of anti-cancer drugs among under-represented groups such as the South Asian population. Situated in South Asia, Sri Lanka is home to a population of approximately 21 million, comprising people from different ethnicities. As per a recent study on genetic variation in drug response and metabolism among the Sri Lankans, there is a significant degree of genetic similarity between the major Sri Lankan ethnic groups and other South Asian populations, whilst being distinct from other European and Chinese (East Asian) populations [7]. The current treatment for most of the cancers in Sri Lanka do not take local pharmacogenomic variants into consideration and most of the guidelines are directly adapted from Western countries with populations that have a distinct genetic profile. Therefore, by exploring the patterns of pharmacogenomic variants affecting the metabolism and response to anti-cancer drugs among Sri Lankans, we aspire to pave the way for personalized treatment approaches to improve the overall outcome of patient care. The present study aimed to explore the diversity of pharmacogenomic variants of genes that are associated with the efficacy and safety of anti-cancer drugs in a South Asian population from Sri Lanka, thereby paving the way for personalized treatment approaches and improve patient outcomes. These findings also bear clinical implications for other South Asian populations who exhibit genetic similarities with the Sri Lankan population.

## Methods

### Identification of pharmacogenomically important genes & variants

Data on pharmacogenomic variants of anti-cancer drugs were obtained from the Pharmacogenomics Knowledgebase (PharmGKB) database (www.pharmgkb.org). PharmGKB is a comprehensive online resource, providing information on how genetic variations influence the efficacy and safety of drugs. PharmGKB contains information on drug- gene interactions, drug pathways, clinical annotations, US FDA-approved drug label annotations, therapeutic guidelines and other relevant data of many drugs. The clinical annotations of the drugs provide information on variant-drug pairs and each clinical annotation is assigned a level of evidence ranging from level 1 A representing the highest level of evidence to level 4 indicating unsupported evidence. For the purpose of our study drug-gene pairs relevant to anti-cancer drugs with Pharm GKB evidence levels 1 A, 1B, 2 A and 2B were selected. Each allele of the gene is assigned a function that corresponds with its phenotype by the Clinical Pharmacogenetics Implementation Consortium (CPIC). Drug-gene pairs with minor allele frequencies of zero within the Sri Lankan population, and the alleles which were assigned ‘Normal function’ were excluded from the analysis, and the remaining variants were included in the study. Pharmacogenomically important genes and variants associated with metabolism of anti-cancer drugs and their functions are listed in Table 1.

**Table 1:** Pharmacogenomically important genes and variants associated with anti-cancer drug metabolism*

Gene	Gene function	Variant (SNV ID^†^)	Drugs	Allele function	Pharmacological effect	Clinical effect	Level of evidence
*CYP2D6*	Tamoxifen is converted to its more active form endoxifen by the CYP2D6 enzyme	CYP2D6*10 (rs1065852)	Tamoxifen	Decreased	Reduced conversion of tamoxifen to active metabolite	Reduced efficacy	1A
*DPYD*	The protein encoded by this gene is a pyrimidine catabolic enzyme and the initial and rate-limiting factor in the pathway of uracil and thymidine catabolism	rs3918290	Capecitabine, Fluorouracil, Tegafur	No Function	Reduced metabolism of the drug in the liver leading to accumulation of the drug in the body	Increased risk of toxicity	1A
		rs56038477	Capecitabine, Fluorouracil	Decreased	Reduced metabolism of the drug in the liver leading to accumulation of the drug in the body	Increased risk of toxicity	1A
*NUDT15*	This gene encodes an enzyme in the Nudix hydrolase superfamily that catalyzes hydrolysis of nucleoside diphosphates, which are a result of oxidative damage, and can induce base mispairing in DNA replication	NUDT15*3 (rs116855232)	Mercaptopurine	No Function	Reduced inactivation of the drug leading to accumulation of the active metabolites	Increased risk of toxicity	1A
*XRCC1*	The protein encoded by this gene is involved in the efficient repair of DNA single-strand breaks formed by exposure to ionizing radiation and alkylating agents	rs25487	Platinum based compounds (e.g. cisplatin, carboplatin, and oxaliplatin)	Decreased	Reduced repair capacity of DNA leading to accumulation of unrepaired DNA lesions caused by platinum based drugs and promote cell death	Increased efficacy	2B
*EPAS1*	This gene encodes a transcription factor involved in the induction of genes regulated by oxygen, which is induced as oxygen levels fall	rs7557402	Sorafenib	Decreased	Reduced VEGF signalling and angiogenesis	Increased risk of toxicity	2B

^*^ Includes only genes and variants where data were available in the Sri Lankan population included in this study; ^†^SNV ID: Single nucleotide variant identifier

### Genotype data & analysis

Frequency of genetic variants of pharmacogenomically important genes associated with metabolism of anti-cancer drugs in the Sri Lankan population were obtained from an existing anonymized database that was derived from 541 Sri Lankans who had undergone exome sequencing at the Human Genetics Unit, Faculty of Medicine, University of Colombo. Prior ethics approval had been obtained for use of this genomic data for pharmacogenomics related studies in the Sri Lankan population [EC-22-012]. The study was conducted in compliance with the Declaration of Helsinki. Ethnicity (Sinhalese, Tamil or Moor) was assigned on the basis of self-reported ethnicity.

Using the Illumina Next Generation Sequencer platforms, whole exome sequencing was performed on the genomic DNA extracted from each individual’s blood. The exon targets were captured using the SureSelect® Human All Exon V6 kits. An in-house bioinformatics pipeline was used for the genetic analysis. The resulting paired end sequences were mapped to the publicly available UCSC hg19/GRCh37 human genome reference sequence construct using the BWA-MEM algorithm. The variant calling procedure was carried out using the Genome Analysis Tool Kit (GATK). The produced variants were annotated using SNP-EFF, with the annotations based on in silico prediction tools, amino acid conservation scores, and population frequencies (1000 Genomes, Exome sequencing project, and internal databases). The resulted variants were interpreted according to the standard American College of Medical Genetics (ACMG) guidelines. Allele counts for each of the genotypes were obtained, and genotype and minor allele frequencies (MAFs) were calculated and tested for Hardy-Weinberg equilibrium (below) and reported with 95% confidence intervals (CI).

Hardy-Weinberg equilibrium: p^2^ + 2pq + q^2^ = 1.

Where p represents the frequency of the dominant allele, q represents the frequency of the recessive allele, p^2^ is the dominant homozygous frequency, 2pq is the heterozygous frequency and q^2^ is the recessive homozygous frequency.

Distribution of the analyzed variants across various population groups and differences in the allelic frequencies of these variants between these populations and Sri Lankans were compared using the χ^2^ test and *p* < 0.05 was considered significant. The MAFs of the variants observed in the present study were compared with MAFs of the variants in different populations extracted from the Genome Aggregation Database - gnomAD v2.1.1 (https://gnomad.broadinstitute.org/).

## Results

The study population comprises 541 individuals. Among them, 54.2% (*n* = 293) were females. The age range in the population varied from 2 days old to 82 years old at the time of sample collection. In the study population, majority of the population, belonged to the Sinhalese group (82.8%; *n* = 448). There were also Tamils, making up 3.7% (*n* = 20) of the population, and Moors, who accounted for 7.0% (*n* = 38). For the remaining 6.5% (*n* = 35) of the population information about their ethnicity were not available in the database. SNVs of the *CYP2D6*, *DPYD, EPAS1, NUDT15* and *XRCC1* genes were studied.

### Genotype frequencies and minor allele frequencies (MAFs) in the overall population

The genotype frequencies and MAFs are shown in Table 2. All observed genotype frequencies were consistent with Hardy-Weinberg equilibrium. The *CYP2D6* (CYP2D6*10) rs1065852 variant had 114 individuals heterozygous and 13 individuals homozygous with a MAF of 12.9% (95% CI: 10.9–14.9). The rs56038477:c.1236G > A variant of the *DPYD* gene had a MAF of 1.5% (95% CI: 0.8–2.2), with 16 individual being heterozygous, while none were homozygous for the variant. Only 13 individuals (2.4%; 95% CI: 1.1–3.2) individuals were heterozygous for *DPYD* rs56038477:c.1236G > A variant, while there were no individuals homozygous for the same variant. The rs56038477:c.1236G > A variant had a MAF of 1.2% (95% CI: 0.5–1.8). A total of 258 (47.7%; 95% CI: 43.5–51.9) individuals were heterozygous for the *EPAS1* rs7557402:c.1035–7 C > G variant, whereas 75 (13.9%; 95% CI: 11-16.7 individuals were homozygous for the variant allele. Overall, the rs7557402:c.1035–7 C > G allele of the *EPAS1* gene had a MAF of 37.7% (95% CI: 34.8–40.6). In the population studied, it was observed that 14.8% (95% CI: 11.8–17.8) of the individuals (*n* = 80) carried the NUDT15*3 (rs116855232:c.415 C > T) variant in a heterozygous state. Whereas 0.9% (95% CI: 0.1–1.7) of the individuals (5 individuals) were found to have the NUDT15*3 (rs116855232:c.415 C > T) variant in a homozygous state. The MAF of the NUDT15*3 (rs116855232:c.415 C > T) was determined to be 8.3% (95% CI: 6.7–10.0). A total of 246 individuals (45.5%; 95% CI: 41.3–49.7) were heterozygous for the rs25487:c.1196 A > G allele of the *XRCC1* gene, whereas 223 individuals (41.2%; 95% CI: 37.1–45.4) were homozygous for variant allele. Overall, the rs25487:c.1196 A > G allele of the *XRCC1* gene had a MAF of 64.0% (95% CI: 61.1–66.8). Genotype frequencies and MAF of these variants are shown in Table 2.

**Table 2:** Genotype and minor allele frequencies of pharmacogenomic variants associated with anti-cancer drug metabolism among Sri Lankans

Gene	Variant (SNV ID^†^)	**Wild-type/** variant allele	Wild-type homozygous, n (%; 95% CI)	Heterozygous, n (%; 95% CI)	**Variant homozygous**, n (%; 95% CI)	MAF^‡^, % (95% CI)	HWE testing	
							X^2^	P value
*CYP2D6*	CYP2D6*10 (rs1065852)	C/T	414 (76.5; 73.0-80.1)	114 (21.1; 17.6–24.5)	13 (2.4; 1.1–3.2)	12.9 (10.9–14.9)	2.264	0.132
*DPYD*	rs3918290	G/A	525 (97.0; 95.6–98.5)	16 (3.0; 1.5–4.4)	0	1.5 (0.8–2.2)	0.122	0.727
*DPYD*	rs56038477	C/T	528 (97.6; 96.3–98.9)	13 (2.4; 1.1–3.2)	0	1.2 (0.5–1.8)	0.080	0.777
*EPAS1*	rs7557402	C/G	208 (38.4; 34.4–42.6)	258 (47.7; 43.5–51.9)	75 (13.9; 11-16.7)	37.7 (34.8–40.6)	0.124	0.725
*NUDT15*	NUDT15*3 (rs116855232)	C/T	456 (84.3; 81.2–87.4)	80 (14.8; 11.8–17.8)	5 (0.9; 0.1–1.7)	8.3 (6.7–10.0)	0.502	0.478
*XRCC1*	rs25487	T/C	72 (13.3; 10.4–16.2)	246 (45.5; 41.3–49.7)	223 (41.2; 37.1–45.4)	64.0 (61.1–66.8)	0.102	0.749

^†^SNV ID (Single Nucleotide Variant Identifier); ^‡^MAF: Minor allele frequencies

### Genotype frequencies and MAFs in different Sri Lankan ethnic sub-populations

Data on genotype frequencies and MAFs in the different ethnic sub-populations (Sinhalese, Tamils and Moors) were available for 506 individuals, and are shown in Table 3. Accordingly, except for the rs25487:c.1196 A > G variant of the *XRCC1* gene between the Sinhalese and Tamil populations, the MAFs of other variants were not significantly different between the ethnic sub-populations of Sri Lanka (Table 3).

**Table 3:** Genotype and minor allele frequencies of pharmacogenomic variants associated with anti-cancer drug metabolism among ethnic sub-populations

Gene	Variant (SNV ID^†^)	Wild-type/ variant	Ethnicity	Wild-type homozygous, n (%; 95% CI)	Heterozygous, n (%; 95% CI)	Variant homozygous, n (%; 95% CI)	MAF^‡^, % (95% CI)
CYP2D6	CYP2D6*10 (rs1065852)	C/T	Sinhalese (n = 448)	337 (75.2; 71.2–79.2)	98 (21.9; 18.1–25.7)	13 (2.9; 1.4–4.5)	13.8 (11.5–16.1)
			Tamils (n = 20)	17 (85.0; 69.4–100)	3 (15.0; 0-30.6)	0	7.5 (0-15.7)
			Moors (n = 38)	32 (84.2; 72.6–95.8)	6 (15.8; 4.2–27.4)	0	7.9 (1.8–14.0)
*DPYD*	rs3918290	G/A	Sinhalese (n = 448)	434 (96.9; 95.3–98.5)	14 (3.1; 1.5–4.8)	0	1.6 (0.8–2.4)
			Tamils (n = 20)	18 (90.0; 76.8–100)	2 (10.0; 0-23.2)	0	5.0 (0-11.8)
			Moors (n = 38)	38 (100)	0	0	0
	rs56038477	C/T	Sinhalese (n = 448)	437 (97.5; 96.1–99.0)	11 (2.5; 1.0-3.9)	0	1.2 (0.5-2.0)
			Tamils (n = 20)	20 (100)	0	0	0
			Moors (n = 38)	37 (97.4; 92.3–100)	1 (2.6; 0-7.7)	0	1.3 (0-3.9)
*EPAS1*	rs7557402	C/G	Sinhalese (n = 448)	174 (38.8; 34.3–43.3)	213 (47.5; 42.9–52.2)	61 (13.6; 10.4–16.8)	37.4 (34.2–40.6)
			Tamils (n = 20)	10 (50.0; 28.1–71.9)	8 (40.0; 18.5–61.5)	2 (10.0; 0-23.2)	30.0 (15.8–44.2)
			Moors (n = 38)	11 (29.0; 14.5–43.4)	19 (50.0; 34.1–65.9)	8 (21.1; 8.1–34.0)	46.1 (34.8–57.3)
*NUDT15*	NUDT15*3 (rs116855232)	C/T	Sinhalese (n = 448)	377 (84.2; 80.8–87.5)	66 (14.7; 11.4–18.0)	5 (1.1; 0.1–2.1)	8.5 (6.7–10.3)
			Tamils (n = 20)	16 (80.0; 62.5–97.5)	4 (20.0; 2.5–37.5)	0	10.0 (0.7–19.3)
			Moors (n = 38)	31 (81.6; 69.2–93.9)	7 (18.4; 6.1–30.8)	0	9.2 (2.7–15.7)
*XRCC1*	rs25487	T/C	Sinhalese (n = 448)	66 (14.7; 11.4–18.0)	200 (44.6; 40.0-49.2)	182 (40.6; 36.1–45.2)	63.0 (59.8–66.1)*
			Tamils (n = 20)	1 (5.0; 0-14.6)	6 (30.0; 9.9–50.1)	13 (65.0; 44.1–85.9)	80.0 (67.6–92.4)*
			Moors (n = 38)	2 (5.3; 0-12.4)	20 (52.6; 36.8–68.5)	16 (42.1; 26.4–57.8)	68.4 (58.0-78.9)

^†^SNV ID (Single Nucleotide Variant Identifier); ^‡^MAF: Minor allele frequencies; *p = 0.03

### Comparison of the variants with other populations

Table 4 shows the distribution of the *CYP2D6*, *DPYD, EPAS1, NUDT15* and *XRCC1* variants across various populations and the differences in MAFs of these variants between these populations and the Sri Lankan population from the present study. The frequency of the *CYP2D6* rs1065852:c.100 C > T variant was significantly lower among Sri Lankans in comparison with Ashkenazi Jewish, East Asian and European (non-Finnish) populations, whilst being similar in frequency to Latino American and African/African American populations. Frequencies of the *DPYD* rs3918290 c.1905 + 1G > A and rs56038477:c.1236G > A variants were significantly higher in the Sri Lankan population than Latino American and African/African American populations. There was a significant variation in the population frequency of the *EPAS1* rs7557402:c.1035–7 C > G variant between the studied Sri Lankan population and individuals from other populations except in other South Asian populations. The frequency of this variant was significantly higher in Sri Lankan population than the African/ African American, East Asian and Latino/ Admixed American populations whereas it was significantly lower in the studied Sri Lankan population than in other populations. The studied Sri Lankan population display significantly higher frequency for the NUDT15*3 rs116855232:c.415 C > T variant than other populations except the East and South Asian populations, where there was no significant difference. In contrast, the frequency of the *XRCC1* rs25487:c.1196 A > G variant in the Sri Lankan population was significantly lower than the African/ African American, East Asian, European (Finnish) and Latino/ Admixed American populations, while it was similar to the frequencies in other populations.

**Table 4:** Minor allele frequencies of genetic variants associated with anti-cancer drug metabolism in different populations compared with the Sri Lankan population

Gene	Variant (SNV ID^†^)	Wild type/ variant allele	MAF^‡^ (p-value^§^)							
			Sri Lankan (present study)	Other South Asian	Ashkenazi Jewish	East Asian	European (Finnish)	European (non-Finnish)	Latino American	African/ African American
*CYP2D6*	CYP2D6*10 (rs1065852)	C/T	0.129	0.168 (< 0.05)	0.246 (< 0.001)	0.541 (< 0.001)	0.113 (NS^*^)	0.222 (< 0.001)	0.153 (NS^*^)	0.125 (NS^*^)
*DPYD*	rs3918290	G/A	0.015	0.003 (< 0.001)	0.007 (NS^*^)	NR	0.024 (NS^*^)	0.005 (< 0.05)	0.002 (< 0.001)	0.001 (< 0.001)
*DPYD*	rs56038477	C/T	0.012	0.0173 (NS^*^)	0.0067 (NS^*^)	0.0001 (< 0.001)	0.0122 (NS^*^)	0.0211 (NS^*^)	0.0045 (< 0.01)	0.0026 (< 0.001)
*EPAS1*	rs7557402	C/G	0.377	0.4066 (NS^*^)	0.4918 (< 0.001)	0.1813 (< 0.001)	0.4409 (< 0.05)	0.4949 (< 0.001)	0.2294 (< 0.001)	0.2676 (< 0.001)
*NUDT15*	rs116855232	C/T	0.083	0.0666 (NS^*^)	0.0036 (< 0.001)	0.1054 (NS^*^)	0.0232 (< 0.001)	0.0035 (< 0.001)	0.0600 (< 0.05)	0.0010 (< 0.001)
*XRCC1*	rs25487	T/C	0.640	0.6482 (NS^*^)	0.6176 (NS^*^)	0.7421 (< 0.001)	0.6883 (< 0.05)	0.6429 (NS^*^)	0.7353 (< 0.001)	0.8547 (< 0.001)

^†^SNV ID (Single Nucleotide Variant Identifier); ^‡^MAF, Minor allele frequencies; ^§^In comparison with Sri Lankan population from the present study; ^*^NS, Not significant

## Discussion

Cancer remains a significant global health burden, with millions of cases diagnosed each year. Anti-cancer drugs play a crucial role in the treatment of cancer. However, individual responses to these drugs vary due genetic heterogenicity affecting the pharmacodynamics and pharmacokinetics of the medications [3]. Previous studies in Sri Lanka have highlighted important pharmacogenomic variants affecting the efficacy and safety of warfarin [8] and statins [9]. There studies have concluded that compared with other super-populations, the frequencies of several variants were significantly different in Sri Lankans, and likely to account for variability in dosage requirements and the occurrence of toxicity due to warfarin and statins. However, there is scarcity of data on pharmacogenomic variants affecting other medicines, especially clinical correlational studies. As a result, at present pharmacogenomic testing is not offered as part of routine care in clinical practice in Sri Lanka.

Currently in Sri Lanka and other South Asian countries, the existing cancer treatments largely overlook the influence of pharmacogenomic variants due to scarcity of data, relying instead on guidelines adapted from Western countries which have populations with distinct genetic profiles. To address this limitation, our study aimed to explore the pharmacogenomic variations that impact the efficacy and safety of anti-cancer drugs in the Sri Lankan population. The study focused on key pharmacogenomically important genes and variants associated with anti-cancer drug metabolism and response, such as *CYP2D6*, *DPYD, NUDT15, EPAS1*, and *XRCC1.* Findings of the study revealed notable differences in the allelic frequencies for some of these genes in the Sri Lankan population.

A previous study on the genetic variants involved in various drug responses and metabolism among Sri Lankan populations was done by Chan et al. and included individuals (*n* = 197) from the three primary Sri Lankan ethnic sub-populations as follows: Sinhalese 24.9%, Tamils 36.5% and Moors 38.6% [7]. This study demonstrated that the Sri Lankan populations showed high levels of similarity at pharmacogenomic alleles. However, in the same study two clinically important SNPs showed moderate/high levels of differentiation [7]. These include *SLC10A2* rs2301159, associated with an increased risk for docetaxel toxicity (twice as common in Tamils and Moors compared with Sinhalese) and *ERCC1* rs3212986, associated with a reduced risk for cisplatin nephrotoxicity (twice as common in Sinhalese and Moors compared with Tamils) [7]. Therefore, it is important to recognize that ethnic sub-population variations may exist, even in a largely homogenous super-population.

Furthermore, in the above study the frequency of *XRCC1* rs25487:c.1196 A > G variant was significantly less compared to the present study (35.1% vs. 62.6% in the present study). However, results from the present study are more comparable with those observed in other South Asian populations. In addition, it is important to highlight that, based on the data published by the Department of Census and Statistics, the overall population of Sri Lanka is composed of approximately 75% Sinhalese, 11% Tamils, and 9% Moors. The present study was conducted in a larger population (*n* = 541) and investigated other genetic variants affecting the efficacy and safety of anti-cancer drugs such as *CYP2D6* (rs1065852:c.100 C > T), *DPYD* (rs3918290: c.1905 + 1G > A and rs56038477:c.1236G > A), *EPAS1* (rs7557402:c.1035–7 C > G), and NUDT15*3 (rs116855232:c.415 C > T), in addition to the *XRCC1* (rs25487:c.1196 A > G) variant.

The present study found that the rs25487:c.1196 A > G variant of the *XRCC1* gene had a MAF of 64.0% in the study population, which was significantly lower than that observed in East Asians, Africans and Latino Americans. This variant allele is associated with reduced capacity to repair damaged DNA which leads to increase in the sensitivity to agents that induce DNA damage, such as platinum based chemotherapeutic agents (e.g., cisplatin, carboplatin, and oxaliplatin). This inference was further strengthened by a meta-analysis done by Hongju Wu et al., which highlights this polymorphism might be a valuable genetic marker for assessing clinical outcomes of platinum-based chemotherapy in the treatment of gastric and colorectal cancer, a relationship that appears to be especially pronounced among Asians, where the polymorphism was associated with reduced response rate to these compounds [10]. A more recent meta-analysis including 23 studies (*n* = 5567) of Asian patients with non-small-cell lung cancer, showed that rs25487 variant of the *XRCC1* gene is a potential marker to predict clinical outcomes of platinum-based chemotherapy in Asian patients [11]. This meta-analysis also demonstrated that the association strength increased with the number of variant alleles, which suggests a gene dosage effect [11]. However, it is important to recognize that individual studies included in the above meta-analysis were predominately from Chinese (i.e. East Asian) populations. However, given the lower presence of the rs25487:c.1196 A > G variant of the *XRCC1* gene in Sri Lankans, they may not exhibit similar clinical outcomes compared to the Western population when treated with platinum-based chemotherapeutic agents which necessitates careful consideration in determining the dosage regimens when adopting treatment guidelines from Western contexts to ensure optimal therapeutic responses.

Tamoxifen is a selective oestrogen receptor modulator that is recommended in the treatment of receptor positive breast cancer in women [12]. Its antagonistic effect of oestrogen at the breast tissue results in anti-tumour properties. Tamoxifen is metabolized by the cytochrome P450 enzymes in the liver to metabolites, such as N-desmethyl-tamoxifen that are active and more efficacious than the parent compound [12]. *CYP2D6* gene and enzyme is important in this metabolic pathway, and variants that leads to reduced or no function results in reduced clinical response [13]. A recent meta-analysis has also shown that CYP2D6*10 polymorphisms alter the pharmacokinetics of tamoxifen in Asian patients with breast cancer [14]. The MAF of the CYP2D6*10:rs1065852C > T reduced function variant in the Sri Lanka population was 12.9%. This suggests that a significant proportion of Sri Lankan patients receiving tamoxifen may not respond optimally to the medication. However, a previous cohort study (*n* = 70), in Sri Lankan patients with hormone receptor positive breast cancer reported a MAF 37.9% [13], likely to be due to differences in sample sizes or patient populations. As noted in Table 4, the MAF reported in the present study is similar to that reported in other South Asians populations (16.8%). Importantly, the observed MAF was significantly lower than in East Asians (54.1%) and Non-Finnish Europeans (22.2%).

Sorafenib is an anti-cancer drug which is used commonly in the treatment of solid organ tumors such as hepatocellular carcinoma and renal cell carcinoma. Its mechanism of action involves inhibiting the tyrosine kinase receptors like vascular endothelial growth factor receptors (VEGFR) and downstream kinases which are involved in tumor growth and angiogenesis [15]. *EPAS1*, an oncogene which is overexpressed in cancer cells in response to hypoxic conditions, serves as a proangiogenic factor in contrast to sorafenib which has anti-angiogenic activity [16]. According to a previous meta-analysis, the rs7557402:c.1035–7 C > G variation in this gene is associated with increased risk of dermatological toxicity in cancer patients undergoing sorafenib treatment [16]. According to the present study, MAF of rs7557402:c.1035–7 C > G in the Sri Lankan population is significantly lower than the European population while it is significantly higher than in the Latino/Admixed American and African/African American populations. This indicates that the Sri Lankan population is likely to show variable response in terms of developing dermatological toxicity when treated with sorafenib, using standard therapeutic regimes based on guidelines adapted from other populations.

Thiopurine drugs such as mercaptopurine and azathioprine are used in the treatment of hematological malignancies like lymphoma and leukemia. NUDT15 (nucleoside diphosphate-linked moiety X-type motif 15) is a key enzyme involved in the metabolism of thiopurine drugs which acts by hydrolyzing the toxic metabolites of the drug [16]. According to CPIC, NUDT15*3 (rs116855232:c.415 C > T) variation of the *NUDT15* gene results in absence of function of NUDT15 enzyme which results in accumulation of the toxic metabolites, indicating that individuals carrying this variant are likely to exhibit drug induced toxicities such as leukopenia, alopecia, hepatic toxicity, pancreatitis and gastrointestinal toxicity, when treated with thiopurine drugs [17]. This inference was further strengthened by studies done in the Caucasian and East Asian populations such as Korean, Japanese and Chinese population [18–21]. These studies strongly recommend genetic testing for *NUDT15* variants before initiating thiopurine therapy to minimize the risk of toxicity in populations with a higher occurrence of the variant. A meta-analysis of *NUDT15* (30 studies) genetic polymorphism on thiopurine-induced myelosuppression in Asians, showed that among *NUDT15* polymorphisms, NUDT15*3 specifically showed a significantly increased risk of early leukopenia (OR 15.31) and early neutropenia (OR 15.85) [22]. However, the studies included were predominately in East Asian populations, with only 2 being based in South Asians. In the present, Sri Lankan population, the MAF of NUDT15*3 (rs116855232:c.415 C > T) variant of *NUDT15* gene is 8.3% which is similar to that of the East Asian population. However, this variant is significantly more frequent in the Sri Lankan population than most of the other different populations, including Europeans indicating that Sri Lankans are more likely to exhibit thiopurine induced toxicities in patients undergoing treatment with thiopurine and may benefit from genotyping *NUDT15* variant before initiating thiopurine therapy to identify the individuals at higher risk and guide personalized dosing strategies to reduce the risk of toxicities.

Fluoropyrimidines such as 5-fluorouracil, capecitabine and tegafur are commonly used to treat colorectal, breast, neck, skin, and stomach cancers. They are antimetabolite drugs which acts by inhibiting the DNA synthesis and repair and thus inducing apoptosis of cancer cells [23]. Dihydropyrimidine dehydrogenase (DPD) is a rate limiting enzyme involved in the catabolism of fluoropyrimidines that is encoded by the *DPYD* gene. Variations in this gene which leads to decreased activity of DPD enzyme increase the risk of hematological, infectious, gastrointestinal, and dermatological adverse effects of these medicines [24]. For instance, the rs56038477:c.1236G > A variation of *DPYD* gene is associated with decreased enzymatic activity according to CPIC guidelines and leads to increased risk of toxicity. A meta-analysis involving studies which had predominantly Western populations (American, and European), concluded that this variant has clinical importance in predicting fluoropyrimidine related toxicity and dosage should be recommended based on the presence of this variant to reduce the risk of developing serious adverse effects when treated with fluoropyrimidines [25]. As per the present study, only 1.2% of the Sri Lankan population has this variant, a figure comparable to that in European population. In addition, the observed MAF in the present study is also comparable to that observed in a 2000 healthy adults from India (1.4%) [26]. However, this variant is found significantly more frequent in Sri Lankan population when compared to other Western populations and East Asian populations.

According to the National Cancer Incidence and Mortality Data Sri Lanka (2020), 37,648 new cancers were diagnosed in the year 2020, of which 39% received chemotherapy using anti-cancer drugs. Hence, it is important to have strategies to optimize patient outcomes while minimizing the drug-related toxicity when using chemotherapeutic agents. The present study identified *XRCC1* (rs25487:c.1196 A > G), *EPAS1* (rs7557402:c.1035–7 C > G), *NUDT15* (rs116855232:c.415 C > T), and *DPYD* (rs56038477:c.1236G > A) variants as pharmacogenomically important in Sri Lankan population and considering local pharmacogenomic data during treatment consideration could be an important strategy to achieve optimum patient care. In addition, the present study may have implications beyond Sri Lanka, as Sri Lankans and other South Asian populations share a similar genetic profile. However, this study is not without limitations. The size of the sample used in this study is relatively small (*n* = 541), warranting further research with a larger sample to strengthen the validity of the results, especially clinical correlation studies, as the present study involved patients/participants who were not using these medicines. It is essential to highlight that the present analysis was done using the exome data, and variants that were not in the exonic regions were not analyzed in this study. In addition, our sample was largely comprised of those from a Sinhalese ethnic background (82.8%), although the conducted sub-population analyses did not reveal any major differences between groups, although with limited numbers from other ethnicities. Furthermore, our results are limited to available SNPs captured in the exome database, and data of several other pharmacogenomic variants associated with efficacy and toxicity of anti-cancer drugs, such as those in genes *CYP2D6*, *CYP2C9*, *NUT15* and *TPMT*, composed of predominantly haplotypic and intronic variants were not available. The study population includes individuals who underwent whole exome sequencing to diagnose rare disorders and the genetic etiology of cancer in their families and does not contain patients who were presently on anti-cancer therapy, preventing direct clinical correlations. However, given the under-representation of pharmacogenomic data from developing countries the information from the present study is important, especially for future planning of clinical correlational studies in similar ethnic populations.

## Conclusions

This study reports the distribution of pharmacogenomically important variants in the genes which affects the metabolism and response of anti-cancer drugs in a South Asian population from Sri Lanka. As per the study, risk of toxicity when treating with sorafenib (rs7557402: c.1035–7 C > G) is probably less in Sri Lankans while it is probably increased when treating with fluoropyrimidines (rs56038477:c.1236G > A) and mercaptopurine (NUDT15*3) in comparison to Western and other Asian populations. Likewise, when treating with platinum compounds (rs25487:c.1196 A > G) Sri Lankans are likely to demonstrate reduced effectiveness when compared to Western and other Asian populations. These findings highlight the importance of incorporating local pharmacogenomic data in the treatment decision-making process to enhance the patient care in Sri Lanka and in other South Asian populations.

## Data Availability

No datasets were generated or analysed during the current study.

## Abbreviations

CI: Confidence Intervals
CPIC: Clinical Pharmacogenetics Implementation Consortium
DPD: Dihydropyrimidine dehydrogenase
FDA: Food and Drug Administration USA
MAF: Minor Allele Frequencies
PharmGKB: Pharmacogenomics Knowledgebase
SNV: Single Nucleotide Variant
